# Efficacy of topical galbanum oil with dry cupping in hospitalized COVID-19 patients: A randomized open-label clinical trial

**DOI:** 10.22038/AJP.2023.21829

**Published:** 2023

**Authors:** Fateme Seydi, Mohammadreza Salehi, Fataneh Hashem-Dabaghian, Fatemeh Emadi, Mohammad Gholami-Fesharaki, Maryam Iranzadasl

**Affiliations:** 1 *Department of Traditional Persian Medicine, School of Medicine, Shahed University, Tehran, Iran*; 2 *Infectious Diseases Department, Imam Khomeini Hospital Complex, Tehran University of Medical Sciences, Tehran, Iran*; 3 *Department of Traditional Medicine, Institute for Studies in Medical History, Persian and Complementary Medicine, School of Persian Medicine, Iran University of Medical Sciences, Tehran, Iran*; 4 *Traditional Medicine Clinical Trial Research Center, Shahed University, Tehran, Iran *; 5 *Department of Biostatistics, Faculty of Medicine, Tarbiat Modares University, Tehran, Iran *

**Keywords:** COVID-19, Cupping therapy, Hijama, Ferula gommosa, Galbanum, Persian medicine

## Abstract

**Objective:**

This study was designed to detect the therapeutic effects of galbanum oil plus dry cupping (a Persian medicine-based method) in hospitalized patients with coronavirus disease-2019 (COVID-19).

**Materials and Methods:**

In this randomized controlled trial, 60 hospitalized COVID-19 patients with positive polymerase chain reaction test (PCR), pulmonary involvement and blood oxygen saturation (SpO2) ≤93 mmHg, were randomly assigned into two groups to take the standard therapeutic regimen alone or alongside cupping and topical galbanum oil (*Ferula gommosa *oleo-gum resin) for 3-5 days. The SpO2 level, the severity of signs and symptoms of patients and laboratory parameters were compared between the two groups.

**Results:**

Fifty-eight patients were analyzed. The SpO2 level changed from 89.27±3.82 to 90.29±3.09 mmHg (p=0.038) in control group, while it increased from 88.74±3.45 to 94.23±2.1 mmHg (<0.001) in galbanum group with a significant difference between the groups (p<0.001). Fever, cough, dyspnea, and anorexia alleviated in the galbanum group more than the control (p=0.003, 0.001, 0.01, and 0.04, respectively). No adverse effects were reported due to galbanum oil and cupping therapy.

**Conclusion:**

Dry cupping with galbanum oil alongside the routine therapeutic regimen could be more effective than the routine therapeutic regimen alone for improving SpO2 level and alleviating fever, cough, and dyspnea in COVID-19 patients.

## Introduction

Corona virus disease of 2019 (COVID-19) has caused a wide range of symptoms from the common cold to fatal respiratory illnesses (Chen et al., 2020). Despite the efforts of scientists to introduce various drugs, there is still no specific treatment for this infection (Consortium, 2021). Available evidence shows the significant role of the immune response in this disease, especially in the lung damage and death. Therefore, immune-boosting and using the antioxidant and anti-inflammatory agents could be effective to reduce the risk or severity of the infection (Mrityunjaya et al., 2020; Shi et al., 2020). 

Cupping therapy is one of the known interventions in Persian medicine, which could be performed with bloodletting (Hijama or wet cupping), or without it (dry cupping). Dry cupping therapy is also done alone or in combination with herbal oil to evacuate waste materials from the body’s organs (Ibn-Sina, 2004). There are numerous studies about the ability of cupping therapy to reduce fever (Liu, 2002). It also has an antioxidant (Tagil et al., 2014), immunomodulatory and anti-inflammatory effects (Ahmed et al., 2005; Zhang et al., 2018), and it can also improve the blood oxygen saturation (Hekmatpou et al., 2013; Ismail et al., 2021). 

Galbanum oil which was used in this study is a Combination of *Ferula gummosa* Boiss essential oil in *Viola odorata –*sweet almond oil as base oil.* F. gommosa* is a plant of the Apiacea family, native to the eastern, central and western regions of Iran. A combination of gum, resin and essential oil (oleo-gum-resin) derived from *F. gummosa *is called “Galbanum” (Mahboubi, 2016). It has antioxidant (Ebrahimzadeh et al., 2011), anti-inflammatory (Mandegary et al., 2004; Moosavi et al., 2015), and also IL-6 inhibitory (Mahboubi, 2016) effects. Its effect on influenza A (H1N1) virus has also been reported in an *in-vitro* study (Moradi et al., 2017). Some of the compounds in this plant (e.g. Δ-Cadinene) have been effective on some surface receptors of the Severe acute respiratory syndrome coronavirus 2 (SARS-Cov2) (Habibzadeh and Zohalinezhad, 2021). 

According to PM, *F. gummosa* is known as “*Barazd*” or “*Ghasni*” and galbamum is called “*Barijeh*”. Barijeh is said to be warm and dry in temperament and is traditionally used to alleviate cough, dyspnea; it also improves the symptoms of asthma (Mahboubi, 2016). 

*V. odorata *(another component in the oil used in this study) is used in Persian medicine and Ayurveda to relieve cough, fever, and upper respiratory tract infections (Mahboubi and Kashani, 2018). Some studies show the effects of *V. odorata* on cough (Qasemzadeh et al., 2015) and fever (Tafazoli et al., 2020). This plant could also be an expectorant and broncholytic drug, due to its saponine compound (Mahboubi and Kashani, 2018). Recently, a clinical trial showed the effectiveness of Viola odorata on cough, myalgia, and headache in patients with SARS-CoV-2. (Mehraban MSA et al., 2023).

Combination of dry cupping with galbanum oil was selected based on the Persian medicine literatures, considering the effects of these interventions in the previous studies, and the main goal of this study was to assess the effects of the cupping therapy with galbanum oil on patients with COVID-19-induced respiratory tract infection. 

## Materials and Methods

**Galbanum oil preparation**

The galbanum oleo gum resin purchased from authentic herbal market, was deposited and authenticated at Herbarium of Faculty of Pharmacy, Tehran University of medical Sciences with voucher specimen number of PMP-1842. To achieve galbanum essential oil by hydro-distillation method, the sample was subjected to the Clevenger apparatus for 4 hr. The yield of essential oil was 10 % (V/W). In the next step, the achieved galbanum essential oil was mixed with almond -violet oil (10%) (Tuba Green Gold Company) as base oil with the ratio of 5:95, and then, stirred to make homogeneous galbanum oil formulation.

**Galbanum essential oil analysis by GC-MS**

Qualitative analysis of isolated galbanum essential oil and almond -violet oil was performed by Gas Chromatography–Mass Spectrometry (GC-MS) method using an Agilent Technologies 7890A equipped with Mass Detector 5975C and capillary column BP20 – SGE (0.25µm*30m). The column temperature was programmed at 60°C for 1 min, then heated to 230°C with the rate of 8°C/min, and left for 5 min. The injection temperature was 280°, and the carrier gas was helium with a flow rate of 1.3 ml/min. The mass range for MS detector was 20-550 m/z with -70 V electron energy. The related peaks were identified based on the NIST 2014 mass spectra library search.

**Sample size**

Taking into account the first type of error of 0.5 and the power of 90% and considering the results of our pilot study, 30 patients entered each group;the following formula was used for comparing the mean blood O2 saturation (SpO2) between the two groups:

$n=\frac{(\sigma_{\mathrm{diff1}}^{2}+\sigma_{\mathrm{d2iff}}^{2}){\left(Z\frac{a}{2}+Z_{\beta }\right)}^{2}}{{\left(\mu_{1\mathrm{diff}}-\mu_{2\mathrm{diff}}\right)}^{2}}$

**Patients and design**

This randomized open-label controlled clinical trial was conducted on 60 real time-polymerase chain reaction (RT-PCR) positive COVID-19 hospitalized patients in Imam Khomeini Hospital Complex (Tehran, Iran) from November to December of 2021.

The research protocol was approved by the Human Ethics Committee of Shahed University of Medical Sciences (Ethical code: IR.SHAHED.REC.1399.108); it was also registered in the Iranian Registry for Clinical Trials (IRCT20201111049349N1).

The inclusion criteria were the age range of 18-80 years, to have symptoms such as severe cough, dyspnea or respiratory rate >24 breaths per minute, existence of pulmonary involvement in the chest CT scan, and SpO2 ≤93%. All the participants completed the informed consent form to enter the study. 

The exclusion criteria were ICU admission, history of gastrointestinal bleeding, pregnancy or lactating, and history of immunosuppressive therapy or chemotherapy in the past month, existence of any histories of allergy to herbal products or skin wounds on the chest.

**Randomization**

 After initial evaluation and obtaining the written informed consent, patients were randomly allocated into two groups; galbanum group or the control group. The block randomization method was used to prepare the random list. In this method, all possible forms of blocks containing four interventions (A, A, B, and B) were specified, and then, were selected randomly using the table of randomized numbers. Finally, a random list consisted of participants’ numbers and their assigned intervention, was created. For concealment allocation, sealed envelopes were used; the participants’ numbers had been written on the external part of envelops, and the group’s name had been written in them. Blinding for patients and assessors was not possible due to the method of intervention. 

**Interventions**

The routine interventions for hospitalized COVID-19 patients (O2 support + Remdesivir 200 mg stat and 100 mg/IV/ daily for 5 days + dexamethasone 8 mg/IV/ daily for 5 to 10 days+ anticoagulant prophylaxis with heparin or enoxaparin) were prescribed for both groups, according to the instructions of the Iranian Ministry of Health at 2020 (Rahmanzade et al., 2020).

The galbanum group also took cupping therapy with topical galbanum oil. The cupping therapy was conducted while the patient was at the sitting position. At first, 2.5 ml of the galbanum oil was rubbed on the back areas of the chest until the oil was absorbed. Then, hot cups were placed behind the chest at the distance of 4 cm from the vertebral spines, fixed at point T4 for one minute, and then sliding (from the areas around the spine, down to the end of the diaphragm pull down) on both sides of the spine for 5 min. Medium-sized hot cup (cup opening width was 5 cm, and cup height was 8 cm with a suction of about 10-15 mmHg) was used in this study. The cupping therapy was performed three times a day for a maximum of five consecutive days at 8 to 10 am, 11 am to 1 pm, and 2 to 4 pm. 

**Outcome measures**

The severity of signs and symptoms of disease including fever, chills, cough, sputum, dyspnea, sore throat, chest pain, myalgia, weakness, headache, dizziness, and rhinorrhea, loss of smell, nausea, anorexia, stomachache, and diarrhea, and the blood oxygen saturation were measured at baseline, and on each day of hospitalization period until the 5^th^ day. 

The severity of each sign and symptom was qualitatively assessed; it was scored as zero=no sign or symptom, one=mild, two=moderate, and three=severe (which limits the daily personal activities such as eating, dressing, or going to toilet or bath). 

The SpO2 was measured daily by Finger Puls Oximetr M130 Caneno without support. In addition, laboratory tests including white blood cell (WBC), blood hemoglobin and platelet count (Plt), erythrocyte sediment rate (ESR) and C-reactive protein (CRP) were measured at baseline and the end of the study (the 5^th^ day). 

The primary outcome was the blood oxygen saturation. The secondary outcomes were the duration and the severity of clinical signs and symptoms, and the changes of laboratory parameters. 

**Table 1:** Compounds identified in the galbanum essential oil (obtained by hydro-distillation) using GC-MS

**No.**	**Retention time (min)**	**Compound**	**Content (%)**
1	1.43	α -Pinene	3.23
2	1.51	β-Pinene	9.77
3	1.6	Sabinene	10.23
4	2.86	o-cymene	1.94
5	5.72	α-copaene	5.7
6	6.44	Germacren D	0.63
7	6.78	Pinocarvone	1.39
8	7.31	Aromandendrene	2.39
9	7.56	Terpinen-4-ol	7.56
10	8.8	Cis-muurola-4(15),5- diene	8.8
11	9.83	Epizonarene	1.07
12	10.161	α -Muurolene	1.48
13	10.95	γ-Cadinene	20.44
14	16.42	Unknown	5.9
15	18.39	Epicubenol	1.34
16	19.06	Guaiol	1.53
17	20.22	T-cadinol	12.03
18	20.78	Bulnesol	1.86
19	21.03	α -cadinol	0.62
Total identified			92.91

**Statistical analysis**

Statistical analysis was performed using R statistical software version 3.1.2 (R Project for Statistical Computing, Vienna, Austria), at a significance level of p<0.05. The qualitative and quantitative variables are reported by frequency (in percent) and mean (±SD), respectively. The Mann–Whitney U test or T-test, and Chi-square or Fisher’s exact were used to compare the studied groups. The within group P-value was also calculated by paired sample t-test, and between-group P-value was calculated by independent sample t-test. In this study, a P-value less than 0.05 was considered statistical significance.

**Table 2:** Chemical constituents of base almond-violet oil detected in GC-MS analysis

**No.**	**Retention time (min)**	**Compound**	**Content (%)**
1	1.63	Decane	8.67
2	1.49	Undecane	14.62
3	2.24	Dodecane	32.09
4	3.34	Tridecane	22.17
5	4.7	Tetradecane	3.32
6	29.58	n-Hexadecanoic acid	2.97
7	31.89	9-Octadecanoic acid,2-hydroxy-1-(hydroxymethyl)ethyl ester	1.96
8	32.9	Oleic acid	7.88
9	33.85	9,12-Octadecanoicacid	2.9
Total identified			95.59

**Table 3:** Baseline characteristics of the two groups

**Variables**	**Level**	**Group**				**p-value**
		**control**		**galbanum**		
		**N**	**%**	**N**	**%**	
Gender	Female	12	40.0%	10	33.3%	0.789
	Male	18	60.0%	20	66.7%	
Age (year)	≤35	4	13.3%	4	14.3%	0.491
	36 – 45	10	33.3%	4	14.3%	
	46 – 55	7	23.3%	7	25.0%	
	56 – 65	4	13.3%	7	25.0%	
	>66	5	16.7%	6	21.4%	
Signs	Fever	16	53%	9	30%	0.115
	Chills	7	23%	4	13%	0.506
	Sputum cough	27	90%	23	77%	0.299
	Dry cough	16	53%	20	67%	0.430
	Shortness of breath	27	90%	24	80%	0.473
	Sore throat	19	63%	15	50%	0.435
	Body pain	13	43%	16	53%	0.606
	Weakness	27	90%	27	90%	0.999
	Headache	15	50%	10	33%	0.295
	Dizziness	7	23%	7	23%	0.999
	Watering	4	13%	3	10%	0.999
	Loss of smell	12	40%	16	53%	0.438
	Nausea	13	43%	11	37%	0.792
	Anorexia	25	83%	23	77%	0.748
	Stomach ache	5	17%	4	13%	0.999
	Diarrhea	5	17%	1	3%	0.195

## Results

**Galbanum oil analysis by GC-MS**

**Figure 1:**
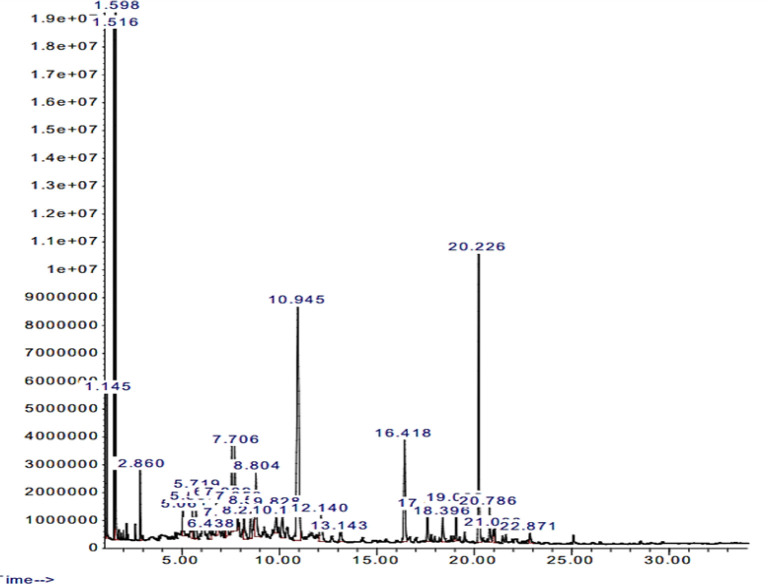
GC-/MS chromatogram of galbanum essential oil obtained by hydro-distillation

**Figure 2:**
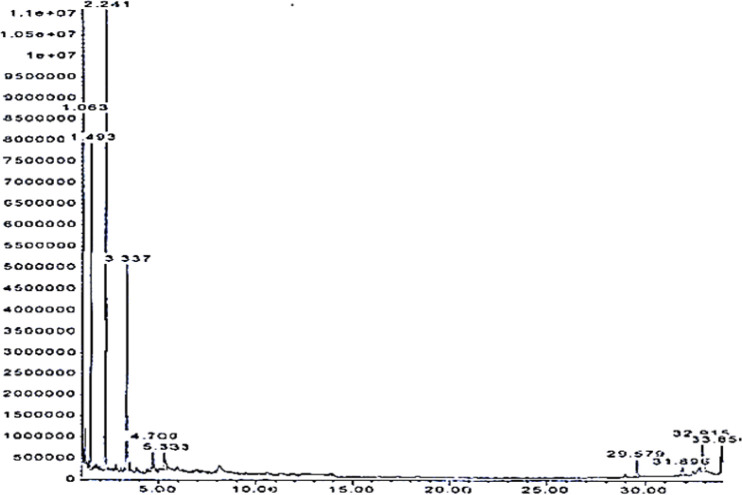
GC-/MS chromatogram of base almond-violet oil

Table 1 and Figure 1 present the chemical constituents of galbanum essential oil based on the GC-MS analysis and related chromatograph, respectively. The major components in galbanum essential oil were T-cadinol, γ-cadinene, sabinene, β-pinene with the percentages of 20.44, 12.03, 10.23 and 9.77, respectively. Result of GC-MS analysis of the base almond-violet oil has been shown in Table and Figure 2**. **

Medical records with high missing data were excluded. One patient in each group passed away and excluded from the analysis. The patients participating in this study included 22 women and 38 men, with the mean age of 51.02±14.56 years; the age range of studied individual was 20- 80 years.

**Figure 3:**
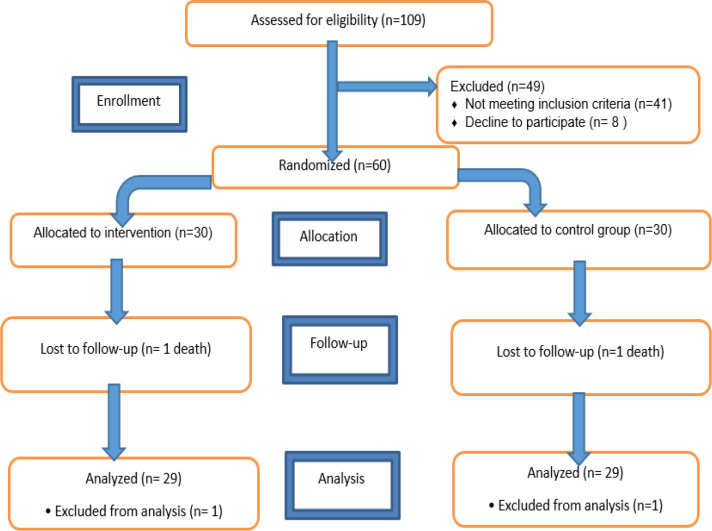
The CONSORT flow diagram of the study

Figure 3 shows the CONSORT (consolidated standards for reporting trials) flow diagram of the participants in this study. Table 3 presents the baseline characteristics of the participants. According to this Table, age, gender and the signs of the disease were not significantly different between groups. The two groups were similar with regard to underlying diseases such as diabetes mellitus, hypertension, and cardiac, liver, kidney, and thyroid diseases (p>0.05). 

**Figure 4:**
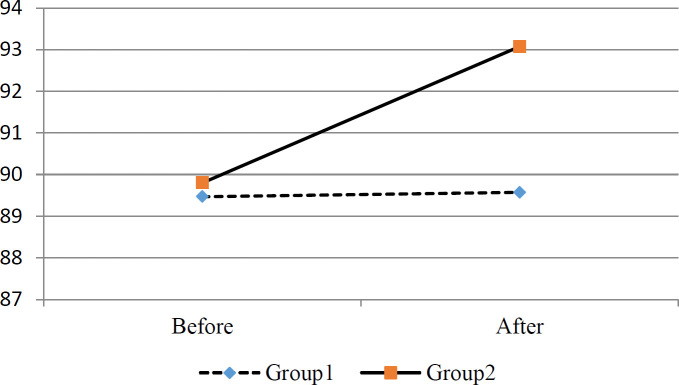
Trend of oxygen saturation in the two groups during the study. Group 1= Control, group 2= galbanum

The SpO2 of pre and post-intervention is reported in Table 4 and Figure 4. According to Table 4, the mean SpO2 level in the galbanum group significantly increased in comparison with the control group. 

The changes of the severity of different signs and symptoms of the disease are presented in Table 5. As shown in this Table, fever, cough, dyspnea, and anorexia alleviated in the intervention group more than the control group. The duration of cough (2.36±1 days in the control group vs. 2.36±1.49 days in the intervention group, p<0.001) and dyspnea (3.13±1.1 days in the control group vs. 2.36±1.21 days in the intervention group, p=0.009) was significantly shorter in the intervention group than the control group. The duration of hospitalization and other symptoms were not significantly different between the groups. 

The amounts of laboratory parameters (CBC, diff, ESR, and CRP) were no significantly different between the groups at baseline and at the end of the study.

**Table 4:** The blood oxygen saturation and its changes during the study

Group					Difference	p-value	
	Baseline		After intervention				
	Mean	SD	Mean	SD		Within groups	Between groups
Control	89.27	3.82	90.29	3.09	1.02	0.038	<0.001
Galbanum	88.74	3.45	94.23	2.10	5.49	<0.001	
P-value	0581		<0.001				

SD: Standard deviation, the within p-value calculated by paired sample t-test and between p-value calculated by independent sample t-test.

**Table 5:** The severity and changes of signs and symptoms during the study

	Group	Mean Diff Day5-Day0	SE	p-value	
				Within groups	Between groups
Fever	control	-0.59	0.10	<0.001	0.003
	galbanum	-0.25	0.06	<0.001	
Chills	control	-0.20	0.07	0.005	0.306
	galbanum	-0.10	0.06	0.101	
Dry Cough	control	-0.67	0.12	<0.001	0.046
	galbanum	-0.96	0.08	<0.001	
Sputum Cough	control	-0.19	0.11	0.084	0.001
	galbanum	-0.72	0.11	<0.001	
Dyspnea	control	-0.97	0.15	<0.001	0.010
	galbanum	-1.50	0.14	<0.001	
Body Pain	control	-0.52	0.10	<0.001	0.209
	galbanum	-0.70	0.10	<0.001	
Weakness	control	-1.25	0.13	<0.001	0.839
	galbanum	-1.21	0.10	<0.001	
Headache	control	-0.57	0.09	<0.001	0.915
	galbanum	-0.58	0.13	<0.001	
Dizziness	control	-0.20	0.06	0.001	0.077
	galbanum	-0.44	0.12	<0.001	
Sweating	control	-0.13	0.04	0.003	0.059
	galbanum	-0.10	0.04	0.01	
Loss of Smell	control	-0.22	0.05	<0.001	0.132
	galbanum	-0.34	0.06	<0.001	
Nausea	control	-0.53	0.09	<0.001	0.181
	galbanum	-0.37	0.08	<0.001	
Anorexia	control	-0.90	0.12	<0.001	0.045
	galbanum	-1.24	0.12	<0.001	
Stomach Ache	control	-0.10	0.07	0.154	0.074
	galbanum	-0.17	0.08	0.035	
Diarrhea	control	0.03	0.10	0.734	0.053
	galbanum	-0.02	0.03	0.439	

## Discussion

The results of the present study showed the significant efficacy of cupping therapy with galbanum oil in improvement of SpO2 and alleviation of cough, dyspnea, fever, and anorexia in the patients with COVID-19 infection. 

There is no evidence to show the effects of dry cupping along with topical form of *F. gommosa *on COVID-19 patients. There is growing evidence of the benefits of cupping therapy in the treatment of various diseases (Aboushanab and AlSanad 2018; Wang et al., 2021). Some studies showed the effects of cupping therapy on pulmonary function. Hekmatpou et al. (2013) showed the effect of wet cupping in comparison with venesection on SpO2 in a sample of smokers with Chronic Obstructive Pulmonary Disease (COPD). In this study, mean SpO2 level increased after wet cupping and venesection, but the increase of SpO2 saturation was higher in the wet cupping group than the venesection group. In addition, the increasing pattern was maintained in the wet cupping group longer than in the venesection group (Hekmatpou et al., 2013). Ismail et al. (2021) also showed that the inter-scapular cupping (both wet and dry cupping) enhanced upper and lower chest expansion, SpO2, and improved blood pressure and pulse rate in the sedentary male smoker students (Ismail et al., 2021). 

Cupping has been presented as a treatment for high fever due to upper respiratory tract infection. In a study, 103 patients with upper respiratory tract infection and fever experienced dry fire cupping on Dazhui (GV 14) and Feishu (BL 13) points for only one session. The body temperature dropped to the normal range and remained at normal level after 14 hours (Liu, 2002). The results of a systematic review indicate that dry and wet cupping are effective on various lung diseases, especially asthma, pneumonia and cough (Joushan et al., 2020). 

In another study, a total of 50 patients suffering from moderate persistent bronchial asthma were evaluated. Cupping therapy was conducted in one group besides the conventional medication, and the results were compared with the conventional medication group. There was clinical improvement in both groups; however, the cupping group showed better responses regarding the pulmonary function tests (Abd al-Jawad et al., 2011). 

Promoting peripheral blood circulation, improving local anaerobic metabolism, improving immunity, modulation of the cellular immune system, regulating humoral immunity and immune-related cytokines, effects on oxidative stress and reducing inflammation are some probable mechanisms of cupping action (Lowe, 2017; Al-Bedah et al., 2019). Zhang et al. showed the effects of cupping on the levels of factors of the inflammatory pathway. It was shown that cupping can reduce the levels of pro-inflammatory factors, increase anti-inflammatory factors, and reduce the production of IL-6 and TNF- α induced by liposaccharides (Zhang et al., 2018).

Recently, a case series on eight COVID-19 patients with ARDS (acute respiratory distress syndrome) showed that warm cupping of the posterior thorax along with the standard management caused a significant improvement in cough, dyspnea, and SpO2 (Karimi et al., 2022). 

According to Persian medicine, “Barijeh” has a warm temperament in the second degree and dry temperament in the third degree. It is used in shortness of breath, cough, and asthma (Mahboubi, 2016). Monoterpenes (including alpha and beta pinene), which are the main components of the essential oil of galbanum inhibit arachidonic acid metabolizing enzymes (cyclooxygenase 1 and 2) and their conversion to prostaglandins and leukotrienes (Lee et al., 2002), and then have an anti-inflammatory effect. In addition, they inhibit IL-6 (Mahboubi, and Kashani, 2018) which increases in the severe type of COVID-19 infection (Muscogiuri, et al., 2020). Therefore, it is likely that the local use of galbanum oil, due to its high penetrating power which has demonstrated its efficacy through topical administration (Emami-Razavi et al., 2016), can be effective in improving respiratory distress, fever and hypoxia in patients with COVID-19. Some of the compounds in Barijeh have been effective on some surface receptors of the SARS-Cov2 virus (Habibzadeh and Zohalinezhad, 2021). It could be important for inactivation of the virus, however further studies should be conducted to approve this effect. 

Tafazoli et al. (2020) showed the antipyretic effect of *V. odorata* oil on children. In this study, 41 febrile children were randomly assigned into two groups. The intervention group asked for rubbing *V. odorata* oil (20 drop) on the peripheral margins of umbilicus. Comparison of this group with the controls showed a significant reduction in the mean axillary temperature without any changes in the control group. In addition, paracetamol usage was significantly lower in *V. odorata* oil group than the control group (Tafazoli et al., 2020). 

Qasemzadeh et al. (2015) reported the effect of *V. odorata* flower syrup on cough in children with asthma. They assigned 182 children aged 2-12 years with intermittent asthma to receive violet syrup or placebo along with a short-acting β-agonist. This study showed that the adjuvant use of violet syrup with short-acting β-agonist can enhance cough suppression in the children with intermittent asthma (Qasemzadeh et al., 2015).

Mehraban *et al.* recently showed that the *V. odorata *L. syrup along with the standard management was significantly more effective than the placebo in alleviating cough, myalgia, headache, and diarrhea in patients with COVID-19 (Mehraban et al., 2023)

Sweet almond which is another component of the oil in the present study that is recommended as a supplementary diet in patients with COVID-19 (Muscogiuri et al., 2020). Some proteins in sweet almonds have the properties of inhibiting angiotensin converting enzyme receptors (Feyzabadi and Pasalar 2016) which has an important role in the severe type of COVID-19 disease (Fang et al., 2020).

The observed effects of the present study could be attributed to the combination of cupping and the topical galbanum oil in total, not to any of these components separately. Further studies should be designed to compare cupping with galbanum oil in COVID-19 or other respiratory tract infections. 

The present study was designed to observe the changes of SpO2 in COVID-19 patients as the primary outcome. The small sample size in this study limited evaluating the changes of other variables. Therefore, conducting studies with larger sample size is suggested to evaluate other outcomes. Another limitation of this study was lack of some standard measurement tools for assessing the signs and symptoms like cough and dyspnea; therefore, performing further studies with standard measurement tools are needed to detect the effect of cupping and galbanum oil on COVID-19 infection signs and symptoms. 

Dry cupping with galbanum oil in combination with routine therapeutic regimen could be more effective than the routine therapeutic regimen; they improve SpO2 level and alleviate fever, cough, and dyspnea in COVID-19 infection. 

## Conflicts of interest

The authors have declared that there is no conflict of interest.
